# Extracellular Matrix Mechanical Properties and Regulation of the Intestinal Stem Cells: When Mechanics Control Fate

**DOI:** 10.3390/cells9122629

**Published:** 2020-12-07

**Authors:** Lauriane Onfroy-Roy, Dimitri Hamel, Julie Foncy, Laurent Malaquin, Audrey Ferrand

**Affiliations:** 1 IRSD, Université de Toulouse, INSERM, INRA, ENVT, UPS, 31024 Toulouse, France; dimitri.hamel@inserm.fr; 2 LAAS-CNRS, Université de Toulouse, CNRS, 31400 Toulouse, France; jfoncy@laas.fr (J.F.); laurent.malaquin@laas.fr (L.M.)

**Keywords:** colon, extracellular matrix, mechanical properties, topography, stiffness, deformability, organoid, scaffold, microfluidic, hydrogel

## Abstract

Intestinal stem cells (ISC) are crucial players in colon epithelium physiology. The accurate control of their auto-renewal, proliferation and differentiation capacities provides a constant flow of regeneration, maintaining the epithelial intestinal barrier integrity. Under stress conditions, colon epithelium homeostasis in disrupted, evolving towards pathologies such as inflammatory bowel diseases or colorectal cancer. A specific environment, namely the ISC niche constituted by the surrounding mesenchymal stem cells, the factors they secrete and the extracellular matrix (ECM), tightly controls ISC homeostasis. Colon ECM exerts physical constraint on the enclosed stem cells through peculiar topography, stiffness and deformability. However, little is known on the molecular and cellular events involved in ECM regulation of the ISC phenotype and fate. To address this question, combining accurately reproduced colon ECM mechanical parameters to primary ISC cultures such as organoids is an appropriated approach. Here, we review colon ECM physical properties at physiological and pathological states and their bioengineered in vitro reproduction applications to ISC studies.

## 1. Introduction

The colon is part of the digestive system, one of the 11 major organ systems of the human body. Its well-known digestive function is to compact the alimentary bolus while absorbing electrolytes and water. However, the colon also plays a critical role in protecting the inner body against external aggressions, whether biological (pathogenic bacteria, virus or parasites); physical (bolus) or chemical (pollutants or food contaminants). This ability to control the uptake across the mucosa while protecting from damage caused by harmful substances is defined as the intestinal barrier function (IBF). A main player of the IBF is a healthy and fully functional epithelium. This epithelium is composed of diverse specialized cell types, all originating from the intestinal stem cells (ISC) [1]. Indeed, ISC ensure the complete renewal of the intestinal epithelial linen within only three to five days, putting great demand on the mechanisms regulating ISC homeostasis and capacities but, also, on the entire tissue cellular organization. Therefore, the colon constitutes one of the most adequate tissues to study stem cells capacities.

The identification of the ISC population during the past decade led to their successful ex-vivo culture, allowing recreating 3D intestinal epithelial mini-organs, namely organoids. This technological breakthrough consists in an isolated intestinal crypt, which includes the ISC, embedded in Matrigel, put in the presence of a culture medium favoring either the ISC renewal or the differentiation process. As a result, these 3D intestinal organoids display all the epithelial cells constituting the colon epithelial linen and represent an excellent model to study the ISC capacity and ability to reconstitute a fully polarized and functional epithelium with all its cell populations (stem cells, transit-amplifying progenitors, enterocytes, enteroendocrine and goblet cells) [2]. This model thus represents an excellent tool to investigate the cellular and molecular mechanisms involved in proliferation and differentiation processes under (patho)physiological conditions.

The ISC surrounding environment, namely the ISC niche, critically controls ISC homeostasis. This niche comprises the supportive mesenchymal cells, fibroblasts being the major population, and the differentiated epithelial progeny, all secreting factors tightly regulating ISC self-renewal, as well as the proliferation and differentiation processes along the crypt. Interestingly, recent findings highlight that physical constraints exerted by the extracellular matrix (ECM) on the epithelium participate in the regulation of the cell phenotypes and behaviors [3]. ECM exerts these constraints on the epithelial cells through specific topography, stiffness and deformability. Under pathological contexts, such as chronic inflammation or cancer, ECM mechanical properties are profoundly modified, participating in the development and progression of diseases such as inflammatory bowel disease (IBD) or colorectal cancer (CRC). Understanding how these ECM constraints affect the intestinal epithelium and which roles they play in the intestinal (patho)physiology is thus of high interest; however, investigating in vivo the impact of ECM mechanical properties on the ISC regulation and the intestinal epithelial linen is challenging. Therefore, using the organoid model represents an excellent alternative to address these questions.

In this manuscript, we review the current knowledge on colorectal ECM physical and mechanical parameters and their role in colon physiology and epithelium integrity, as well as their evolution during colorectal pathologies, and we discuss how organoids can help increase this knowledge.

## 2. Colon Epithelium and Extracellular Matrix Interactions

### 2.1. The Colon Epithelial Cell Populations

**Figure 1:**
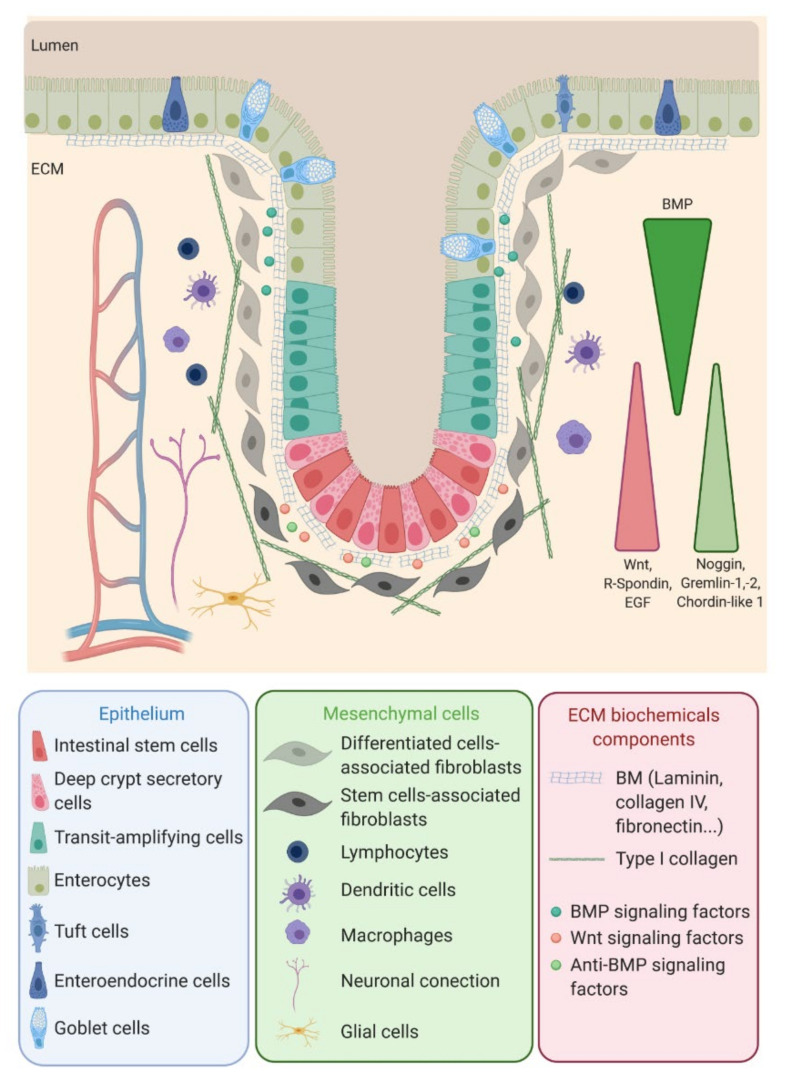
Intestinal stem cells (ISC), located at the bottom of the crypt, give rise to transit-amplifying (TA) cells that migrate along the crypt during their differentiation while reaching the surface. ISC regulation, as well as the proliferation and differentiation processes, are mediated by signals emanating from the surrounding mesenchymal cells, close-by epithelial cells and the interaction of the epithelial cells with the basal membrane. EGF: Epidermal Growth Factor, ECM: extracellular matrix, BMP: bone marrow protein and BM: basement membrane.

The colon epithelium is a single layer of polarized cells, their basal pole leaning on the basement membrane and their apical pole facing the lumen (Figure 1). Observed from the lumen, the colon epithelium is flat, contrary to the small intestine epithelium that displays villi, finger-like protrusions that project into the lumen of the gut. However, like the small intestine, the colon presents invaginations called crypts of Lieberkühn. The peculiar architecture of the intestinal epithelium allows compartmentalization of ISC at the bottom of the crypts and differentiated cells at their top (Figure 1) [1]. The intestinal epithelium is subjected to a fast renewal, occurring within three to five days, to maintain its integrity and optimal function. Constituted up to 80% by absorptive enterocytes, the colon epithelium also contains goblet cells (18%) secreting the mucus, tuft cells (0.4%) playing an antiparasite function and enteroendocrine cells (1% of the cell population) secreting diverse hormones controlling the intestinal motility and secretions and colon peristalsis but, also, visceral sensation, appetite or pancreas exocrine functions. Contrary to the small intestine, Paneth cells are absent from the colon epithelium, where deep crypt secretory (DCS) cells function as their colon equivalent [4]. Differentiated cells originate from transit-amplifying (TA) cells, progenitor cells that are characterized by a rapid proliferation rate before migrating along the crypt and differentiating while reaching the luminal surface. The TA cells rise from the ISC.

ISC function and behavior are precisely regulated by their niche. Along the crypt axis, the expression profiles of signaling pathways in epithelial cells along the intestinal tract, as well as the crypt-villus axis, have been studied for the past decade in the small intestine and colon of pigs [5], mice [6], rats [7] and humans [8] and recently reviewed by Wang et al. [9]. Using complementary DNA (cDNA) microarrays, Kosinski et al. were able to discriminate key pathways involved along the human colon crypts [8], corroborating results obtained in mice small intestines by Mariadason et al. two years before [6]. Logically, at the bottom of the crypt, the expression of genes regulating the cell cycle or, more specifically, the ISC and progenitors (Wnt, R-spondin, HedgeHog (Hhg) and Epidermal Growth Factor (EGF)) are increased, while those inducing apoptosis are downregulated. On the other hand, pro-differentiative bone marrow protein (BMP) pathways are preferentially expressed at the top of the crypt. Interestingly, BMP antagonists such as Noggin, Gremlin 1, Gremlin 2 and Chordin-like 1 are only expressed by the surrounding stromal cells, myofibroblasts and smooth muscle cells at the bottom of the crypt. The leading role of the epithelial-mesenchymal dialogue in intestinal epithelium differentiation and crypt-villus formation has already been demonstrated using a co-culture of intestinal epithelial cells and mesenchymal cells [10,11]. This suggests a compartmentalization not only of epithelial cells but, also, of mesenchymal cells through specific gene expression profiles along the crypt axis. This compartmentalization is indeed a key determinant in intestinal homeostasis.

### 2.2. In Vitro Culture of the Colon Epithelium

Culture of 2D intestinal epithelial cells has proven its worth, allowing to study intestinal barrier functions using Caco-2 cells cultured on Transwell plates or mucus secretion using HT29-MTX cells differentiated in goblet cells using methotrexate. Caco-2 cells are also commonly used to study epithelial differentiation [12] and migration [13]. Other human colon cancer cell lines, such as SW480, have helped to investigate invasive and metastatic potentials and decipher tumorigenic capacities of subpopulations through in vitro and in vivo analyses [14,15,16], therefore providing clues of genetic implication in the tumoral aggressiveness of the colon cancer.

However, colon 2D cultures have limitations, especially to study tissue architecture and spatial organization of the cells. Therefore, the development of 3D structures, such as spheroids or organoids, has enhanced the suitability of scientific research by restoring cell–cell and cell–matrix interactions. While spheroids are generally generated using established cell lines and, mostly, tumoral cell lines, organoids are created from stem cells, allowing the development of multiple cell lineages, recapitulating the full organ (patho)physiological parameters. Moreover, contrary to spheroids, cells growing into intestinal organoids are able to develop as single-layered epithelium surrounding a central lumen. Colorectal organoids can be obtained from primary intestinal stem cell explants from mice [2] or humans [17] and are now widely used to better understand the intestinal physiology and pathology [18,19,20,21]. Indeed, intestinal and colonic cell lines are mainly derived from tumor samples, thus not allowing studying physiological mechanisms or elucidating those implicated in the switch from physiological towards pathological cell phenotypes. The development of organoids and the access to tissues samples from either healthy/normal or pathological tissues at different stages of a disease (inflammatory bowel disease (IBD) or cancer), sometimes even from the same patient, have changed the game. Thanks to the better relevance of this model, the use of colorectal organoids participating in cancer initiate understanding [22], improve the drug screening for CRC [23] and will lead to the expansion of personalized medicines [24]. Organoid cultures have now been obtained for different organs, allowing research progress on ovarian cancer [25], liver cancer [26] or breast cancer [27], for instance. Interestingly, some CRC samples may not be cultured as organoids [28]. Through RNA sequencing, a set of genes differentially expressed in those samples compared to organoid-forming samples have been found. Mainly, genes implicated in the regulation of immune and inflammatory responses appear to be significantly upregulated in nonforming tumors, suggesting that stromal factors are key determinants for ISC and cancer stem cell maintenance, phenotype regulation, survival and tumor growth.

3D in vitro studies have produced remarkable results. However, a limitation of colorectal organoids cultured from 3D into hydrogels such as Matrigel is that their lumen are not easily accessible to explore lumen content interactions with the epithelium. The only way to overcome this issue is to inject the material of interest (bacteria, toxins, etc.) into the organoid lumen, altering then the epithelial linen. Remarkably, intestinal epithelial cells originated from human-induced pluripotent stem cells (hiPSC) and grown in 3D as organoids have successfully been plated into 2D to form a fully functional permeable membrane in order to study the drug absorption and availability through the intestinal barrier [29]. Actually, isolated primary ISC can be grown as 3D organoids, then plated into 2D while keeping their cellular organization [30]. Indeed, pairing primary organoid cultures, grown in 2D, together with the development of new devices mimicking the 3D architecture of the intestinal and colonic epithelium, as well as other ECM mechanical properties such as topography, stiffness and deformability, may represent interesting tools to address the intestinal epithelial barrier function or tissue mechanobiology and architecture.

## 3. Colorectal ECM: Composition and Mechanical Properties

### 3.1. ECM Composition

**Figure 2:**
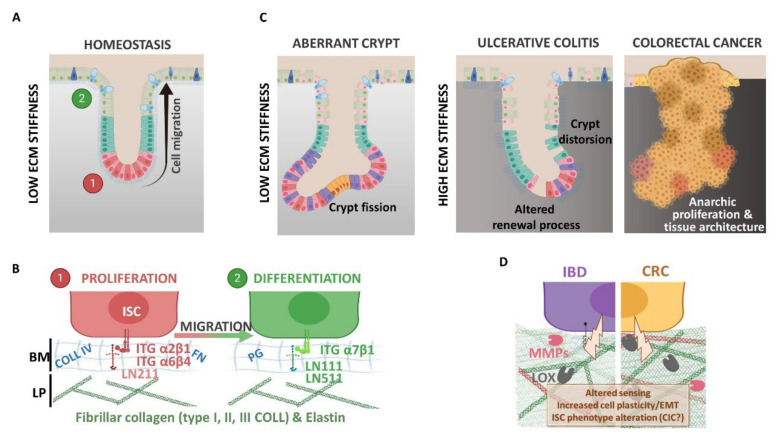
(**A**,**B**) Extracellular matrix (ECM) and epithelial cell phenotype regulation under physiological context. Colonic epithelial cells interact with the basement membrane (BM) composed of collagen type IV (COLL IV), fibronectin (FN), several proteoglycan (PG) and laminin (LN) subtypes. The BM allows epithelial cell migration from proliferating to differentiating compartments along the crypt axis. Different LN isoforms are expressed in these two compartments: LN211 is expressed at the crypt bottom near the proliferative cells (ISC and TA cells), whereas LN111 and LN511 are mainly found within the differentiation compartment. The differentiated cells sense ECM components with different integrin (ITG) isoforms. (**C**,**D**) Extracellular matrix (ECM) and altered epithelium contexts. In the aberrant crypt or during inflammatory bowel disease (IBD) and colorectal cancer (CRC), the epithelium displays several alterations, like a tissue architecture deformation (i.e., crypt fission or distortion), and increases the ECM rigidity support (inflammation and cancer). In fact, the ECM network undergoes high remodeling during pathology compared to physiology: (i) increased fibrillar collagen deposition (COLL I and III) and crosslinking via lysyl oxidase enzymes (LOX) that modify colonic tissue stiffness and (ii) basement membrane disruption and ECM free fragments releasing due to metalloproteinase degradation (MMP, like MMP2 and MMP9). ECM processing affects epithelial cell behaviors like proliferation, stemness, epithelia-mesenchymal transition (EMT) or survival, contributing to disease progression and favoring cancer-initiating cell (CIC) phenotypes.

ECM is another actor of the ISC niche. As in most organs, colorectal ECM has two compartments: the basement membrane (BM) and the interstitial matrix, also called lamina propria. Colon BM is mainly composed of type IV collagen, laminin 111 and 211, elastin and proteoglycan, as well as proteins also present in the lamina propria, such as tenascin-C and fibronectin, organized in a thin layer (100–300 nm) and synthesized by both mesenchymal and epithelial cells [31]. The BM supports the epithelial linen, forming a barrier between the epithelium and the stroma. Stromal cells (fibroblasts, neurons, glial cells and immune cells) and vessels are inserted in the interstitial matrix; the major components are type I, II and III collagens and elastin [32], providing the tissue 3D scaffold. The BM and interstitial composition and stiffness vary depending on the physiological or pathological contexts (Figure 2).

The differential expression profiles of BM molecules along the crypt-villus axis in the human small intestine were described more than twenty years ago [33] and are summarized in Table 1. As an example, through indirect immunofluorescence, Beaulieu et al. showed that the A-chain, corresponding to the α1 variant of the heavy chain of laminin molecules, is predominantly associated with the differentiated cells on the villi, whereas the M-chain, represented by the α2 variant of the heavy chain, is restricted to the crypts [34]. The interactions between the epithelial cells and the BM components are mediated by integrins, which also display differential expression profiles along the crypt-villus axis in the human small intestine [33,35]. Those interactions are involved in the ECM bioactive roles. Indeed, aside from its structural role, the ECM composition can regulate many biological pathways, including adhesion, proliferation or morphology [36], and has been shown to be determinant for the differentiation of stem cells, such as liver progenitor cells [37], induced pluripotent stem cells [38] and, also, colon epithelial cells [12]. Cell–ECM interactions via integrins are necessary to promote survival [39,40]. In the gastrointestinal (GI) tract, the exfoliation of the differentiated cells by anoikis, a cell death induced by the loss of interaction with the basal membrane, participates in the maintenance of the epithelium renewal. Integrin engagement is necessary to inhibit this programmed cell death. For example, α2β1 and α5β1 suppress anoikis in undifferentiated cells, while α3β1 suppresses anoikis only in differentiated cells [41]. The role of ECM in cell adhesion and signaling through integrin receptors has received great interest [35,42]. Besides, for more than 20 years, investigations have highlighted the central role of the ECM in tissue physiological, as well as pathological, states [43,44] (Figure 2).

Under pathological conditions, ECM remodeling correlates with fibroblasts activation and proliferation, the recruitment of mesenchymal and immune cells near the epithelium and alteration of the epithelial cell phenotypes.

The impact of this remodeling, including ECM biochemical and mechanical property alterations, on the disease process is a concept that is now well-accepted [45,46]. During colorectal pathologies, especially during IBD and CRC, an intense remodeling of the ECM is observed [47,48,49]. IBD is a term regrouping two types of GI disorders: Crohn’s disease (CD) and ulcerative colitis (UC). They are defined by chronic cycles of inflammation and wound healing of the GI tract, leading to intestinal fibrosis, strictures and stenosis. However, while CD affects the entire GI tract, from the mouth to the anus, UC is restricted to the colorectal segment. Deciphering ECM profiles and understanding its remodeling in IBD subtypes could lead to a better knowledge of the inflammatory processes involved and would provide optimized disease diagnostics and treatments. Indeed, it has been found that some proteins such as matrix metalloproteinase (MMP)-7 [50], matrix metalloproteinase degraded biglycan (BGM) and citrullinated and matrix metalloproteinase degraded vimentin (VICM) [51] can be used as biomarkers for differentiating CD and UC patients, highlighting that the ECM turnover profiles are different between UC, CD and irritable bowel syndrome. CRC primarily arises from genetic and epigenetic alterations occurring in epithelial cells, resulting in an initial benign polyposis that further develops into adenoma and then carcinoma, invading the interstitial matrix [52]. Those processes require the degradation of the existing ECM through the production and activation of MMP and adamalysins (ADAM). For instance, upregulation of the expression and/or activity of MMP-2 and MMP-9 is observed in both IBD and CRC samples [53,54,55]. ADAM10 has been shown to be overexpressed in CRC later stages [56] and to promote metastasis to the liver in mice xenografted in the spleen with HCT116 human CRC cells that overexpress this protein [57]. The expression of ADAM17 and ADAM15 is increased in intestinal epithelial cells of inflammatory tissues [58,59]. To go further, a proteomic approach has allowed the establishment of an ECM protein signature of human primary colon carcinomas [60]. A subset of ECM glycoproteins, such as thrombospondin-2 (THBS2), ECM regulators including lysyl oxidase homolog 2 (LOXL2) or procollagen-lysine,2-oxoglutarate 5-dioxygenase 1 (PLOD1), and affiliated proteins such as MUC13 have been specifically identified in colon cancer samples. Among those proteins are also MMP-2, MMP-9 and ADAM10, as previously described.

Through matrix protease expression, cells disrupt ECM fibers in order to produce new ones. Type I collagen is the main component of this new microenvironment, as its expression increases with the different stages of CRC at the expense of type IV collagen [61]. This new balance has a positive influence on cancer cell progression in vitro and in vivo. Moreover, the expression of lysyl oxidase (LOX), a secreted collagen crosslinker, is also positively correlated with CRC progression [62]. Interestingly, type I collagen production is increased in human inflammatory intestinal tissues [63,64], and LOX expression is upregulated in rat colitis [65]. Therefore, one can propose that those expended depositions could contribute to the increased risk of CRC development in IBD patients [66] by creating a context that will favor tumorigenesis due to an alteration of the ISC niche that may lead to a loss of ISC homeostasis.

### 3.2. ECM Mechanical Characteristics

#### 3.2.1. Topography

##### Physiological Description and Pathological Evolution of Colorectal ECM Topography

Topography is the study of the shape and the features of a surface. Not to be confused with topology, that is a mathematical study of properties of an object preserved under continuous deformations, stretching and squeezing.

If colon topography, i.e., a crypt scaffold of colorectal ECM, is well-known, very few studies were interested in determining accurate crypt dimensions. Human normal colonic crypt dimensions, evaluated using scanning electron microscopy (SEM), are around 50 µm in diameter and 300–400 µm in length [67]. Differential interference contrast (DIC) microscopy on dissected human colonic crypts confirmed the size as measuring 433 µm in height and 73.5 µm in diameter [68]. Crypts morphology is a key determinant of the intestinal integrity and function. Due to chronic inflammation, fibrosis or anarchic cell proliferation, architectural crypt distortions are observed in both CRC and IBD. This phenomenon is called “aberrant crypt foci” (ACF) or “corrupted colonic crypts” (CCC), respectively [69,70,71,72]. Alterations of the crypt morphologies, accompanied by the fragmented mucus layer, have been described in CRC samples since the 1980s [73,74]. Histologically, ACF are considered as the earliest morphological lesions observed in the sporadic CRC process. Computer modeling supports this observation by demonstrating that an activating mutation within the Wnt pathway mediators, such as mutations of the *adenomatous polyposis coli* (*APC*) gene, which are the first genetic alterations observed in 80% of sporadic CRC, can induce colonic epithelium deformation, budding and crypt fission [75].

Ultrastructural consequences of IBD have similarly been assessed in adult patients in the 1980s [76,77] and in children [78]. In most cases, a decreased number and an irregular spacing of the colon crypts are observed in UC patients. Some patients also present a dysplasia, resulting in a marked variability in the size and shape of the surface epithelial cells with enlarged morphologies. A SEM analysis on the large bowel mucosa of children with CD or UC revealed that, while the colons of CD patients mainly presented an enlargement of the extrusion zones with a regular and conserved pattern of the crypt openings, the mucosa of UC patients presented a decreased number of crypt openings associated with crypt distortion, abscesses and a filamentous mucus [78]. Phenotypes of those crypt distortions, or CCC, observed in UC are various, comprising, for example, asymmetric lateral fission and dual or three-foiled corrupted fission [71,72]. Interestingly, the UC patient risk of developing colitis-associated CRC is higher than CD patients.

##### Reproducing Colorectal ECM Topography

In addition to providing protection for ISC and facilitating the formation of factor gradients, one has to wonder if ECM invaginations may have other impacts on the intestinal physiology. Particularly, are crypt dimensions and curvature determining the regulation of the tissue functions? Computer modeling has proposed that cellular adhesion and migration are influenced by substrate curvature, affecting the cytoskeleton force networks and cell shape depending on the degree of bending [79,80,81]. Those in silico modeling observations were confirmed by in vitro experiments using renal epithelial cells [82] and mesenchymal stem cells (MSC) [83]. Curved substrates increase the expression of polarization markers, ZO-1 and NaK-ATPase α1, on renal epithelial cells compared to flat surfaces [82]. Besides, MSC spontaneously differentiate toward adipose lineage with a uniformly spindle-shape when grown on 500-µm micro-glass embedded in polyacrylamide gel compared to flat plates [83], confirming that convex substrates stimulate cell differentiation. Altogether, these studies suggest that ISC located at the bottom of the crypt could sense the degree of bending, thus impacting cell–cell or cell–BM interactions and cell orientation into either self-renewal or differentiation pathways.

To verify this hypothesis, new devices mimicking the topography of the colon epithelium have been developed to decipher peculiar topography implications on intestinal cell behavior. Since the past decade, a large number of natural and/or synthetic materials, as well as technologies, have been used to produce those devices, as recently reviewed [84]. The challenge resides in the dimensions of the specific colon topography, which have to be relevantly reproduced. Interestingly, it seems that most of the used materials and technologies are able to produce such structures through more or less complex multistep manufacturing processes (Table 2). Regarding the colon topography, Wang L et al. created a Sukhoi SU-8 mold by photolithography with a villus pattern of 120 µm in height and 50, 100 or 500 µm in diameter and then built a negative replicate of this mold in polydimethylsiloxane (PDMS), leading to the formation of crypt-like structures coated later with ECM proteins [85]. Another approach is the one of Wang Y et al. that used UV-exposed multilayers of a photoresist epoxy to produce a crypt mold generating a negative PDMS stamp of 430 µm in height and 125 µm in diameter [86]. This stamp then enabled the creation of a crypt footprint in a collagen scaffold placed on a Transwell insert. Very recently, Lutolf’s team published a new gut-on-chip device based on a simple method of fabrication, using a Matrigel and collagen I mixture embedded in a microchip and laser-ablated to reproduce mouse small intestine crypt geometry [81]. For intestinal topography, i.e., villus or crypt-villus scaffolds, Creff et al. developed a specific biocompatible hydrogel photopolymerized to reproduce crypt-villus dimensions [87]. In contrast to this one-step process, March’s team elaborated a manufacturing process using laser ablation to build an array of 500-µm deep holes in polymethyl methacrylate (PMMA), which served as a mold for a PDMS stamp [88,89,90,91]. Then, agarose replicas were built using this stamp to allow the creation of a final scaffold either in collagen, poly-lactic-glycolic acid (PLGA)-porogen or poly(ethylene) glycol diacrylate (PEG-DA).

##### Cell Culture on In Vitro Scaffolds Mimicking Colorectal ECM Topography

Regarding cell lines, intestinal cell lines such as Caco-2 are often used as a proof of concept for the reliability of the device. Interestingly, those devices can be used to include other aspects of intestinal physiology, such as microbiota [91] and vascularization [94]. For example, it has been observed in differentially located pathogen bacteria along the villus, according to the state of differentiation of Caco-2 cells [91]. Moreover, a 3D bioprinting approach was used to shape a unique villus containing human umbilical vein endothelial cells (HUVECs) in the core and Caco-2 cells at the periphery, recreating a vascularized villus [94].

Considering primary cultures, 3D organoids generated from tissues isolated from mice and humans have successfully been cultured in 2D onto micro-well scaffolds for several days. Small intestinal crypts isolated from mice have been maintained for seven days on villous-like substrates [89]. In this model, Paneth cells were localized at the bottom of the crypt-like structures within the scaffold, while goblet cells were found along the villus, suggesting an accurate spatial organization of the differentiated cells. Interestingly, Lutolf’s team successfully cultured mouse primary proximal ISC up to one month on a gut-mimicking device, forming an epithelium reconstituting all the intestinal cell types [95]. To go further, human primary colonic cells were able to colonize and survive for 32 days on a collagen-based crypt-like scaffold [86]. Remarkably, chemical gradients from the luminal and the basal compartments allowed the formation of polarized crypts with stem cells at the bottom and differentiated cells at the top of the crypt. The data demonstrated that cells at the crypt surface were derived from proliferative cells located at the bottom of the crypt. Therefore, the challenge of recreating a colon topography coated with a complete monolayer epithelium from the ISC has been raised.

Many articles have been published regarding the making of original devices supporting the development of a differentiated colon epithelium. However, deciphering the impact of the topography itself on an ISC phenotype remains to be understood. How are parameters such as depth, diameter and bending instrumental for ISC regulation? Wang L. et al. showed that a micro-well scaffold designed to recreate physiological intestinal crypt dimensions slowed down Caco-2 spreading and/or proliferation [85,96]. Interestingly, the lag phase before cell expansion was longer on 50-µm diameter invaginations compared to 100 µm and 500 µm [85]. Further analyses have been made using other type of stem cells, such as human MSC [97] or human embryonic stem cells [98]. Both showed that peculiar topographical patterns such as lines, pillars or wells of different dimensions determine the differentiation towards osteogenic and adipogenic or neuronal and glial lineages, respectively. Thus, these results highlight the potential role of the intestinal crypt shape in the regulation of the cell behavior and fate. However, much remains to be done. Three-dimensional organoids grown in 2D on micro-well scaffolds mimicking healthy or pathological crypts could help elucidate the fundamental key processes in ISC regulation and therapeutic approaches.

#### 3.2.2. Stiffness

##### Physiological Stiffness of Colorectal ECM and its Evolution in Pathological Contexts

Stiffness is the measure of resistance when a force is exerted on an elastic object; the force needed to contort the object defines it. Considering biological tissues, mechanical determination of the matrix stiffness is expressed as the elastic modulus (EM, Young’s modulus). The EM has been assessed for the human brain, skin, breast and bone marrow tissues, among others [99,100,101,102,103]. Focusing on the human colon, measuring stiffness on cryopreserved normal colon sections by the atomic force microscopy (AFM) approach determines an EM at 0.8 kPa [104], using a tactile sensor on fresh longitudinally opened normal colons measures, an EM of 0.9 kPa [105], while using a custom-built multiscale indenter on ice-conserved normal colons detects an EM at 0.7 kPa [64] (Table 3). Interestingly, different colon samples preparations and different instruments lead to the same range of mean/median Young’s modulus. However, when considering the human small intestine, discrepancies appear. Using a tissue elastometer, Johnson et al. measured median EM of fresh human normal small intestine samples at 2.6 kPa [63], when Stewart et al. obtained 0.6 kPa using their own custom-built instrument [64]. Those results highlight that, despite the homogenous values obtained on colon samples, there still is the need to standardize the EM measurement procedures on tissues. Another way could be to include the measurement of control samples whom the EM already know. Yet, variability is also visible within the same study, as illustrated by Kawano et al. Using the same instrument and protocols to measure the stiffness of multiple normal colon tissues, they obtained EM ranging from 0.3 kPa to 7.3 kPa [105]. This indicates that interindividual variability will always have to be taken into consideration.

Degradation of the initial ECM components and secretion of others by stromal cells, mainly fibroblasts, and epithelial cells during colorectal pathogenesis induces the remodeling of the ECM organization and alters the tissue stiffness. Indeed, the inner matrix mechanical properties are the result of three main factors: the intrinsic elastic properties of the matrix molecular constituents, their density and their spatial organization. During inflammation and cancer, abnormal matrix fibrillary proteins production and reorganization of their spatial orientation increase the matrix stiffness [104]. As a result, Johnson et al. measured the stiffness of the small intestines of CD patients at 16.7 kPa [63], six-fold higher than in healthy small intestines (Table 3). Intriguingly, Stewart et al. measured the inflammatory ileal tissue at 0.99 kPa, significantly higher than a normal sample but much lower than Johnson’s result [64]. As discussed above, the discrepancy between studies certainly mainly comes from the tissue preparation and measurement methods used. When studying inflammatory tissues, the level of inflammation and ECM remodeling are certainly not identical from one injured area to another and depend on the degree of development of the pathology. Therefore, the standardization of sample preparation appears to be unrealistic. A good illustration is the report by Kawano et al., in which they measured the elasticity of human CRC samples and ranked the results depending on the CRC stage [105]. As expected, the EM and interindividual variability significantly increased with the CRC progression. As a matter of interest, the matrix stiffness is positively correlated with collagen fibers and the α Smoooth Muscle Actin (αSMA)-positive area, arguing for a substantial influence of the cancer stroma on the ECM mechanical properties.

##### In Vitro Modulation of Substrate Stiffness to better Mimic Colorectal ECM Rigidity

One challenge of crypt-villus-like scaffolds is to accurately reproduce the ECM mechanical properties. The stiffness of natural and synthetic polymers can be very diverse, spanning from Pa to GPa, allowing the reproduction of diverse biological tissues. Elasticity of the hydrogels is dependent on the concentration of the main component and the level of the crosslinker. The elasticity of the intestinal and colic tissues at the physiological, as well as pathological, states has been measured between 1 to 68 kPa. However, few teams that have developed 3D crypt-villus scaffolds were interested in the elastic modulus of the material used, thus confirming the biological relevance of their device. The most frequently used hydrogel to produce design scaffolds with controlled stiffness is polyacrylamide (PA) gels [106,107,108]. PA gels consist of a mixture of a monomer, acrylamide and a crosslinker, bis-acrylamide, which ratio can easily be controlled in order to produce polymers with tunable stiffness. It is now commonly used to build a 2D matrix with accurate stiffness [109,110,111]. Recently, Comelles et al. used PA hydrogels with independently controlled topography and stiffness to grow mouse intestinal epithelial cells [106]. However, the designed patterns of 5–10 µm are significantly smaller than the intestinal crypts, and the stiffness varying from 3 kPa to 145 kPa is rather representative of a pathological state of the colon epithelium than a physiological one. Thereby, much remained to be done in order to produce in vitro relevant devices. Tunable properties and ease of shape of the PA hydrogel make it a good lead to take up this challenge. Besides, PA gels are permeable to small molecules and can be coated with ECM proteins to simulate the basal membrane. Finally, primary stem cells, such as iPSC and MSC, have been successfully grown on 2D and 3D PA hydrogels [38,112,113]; therefore, ISC should successfully be cultured as well.

##### Cellular Effects of Variable ECM Stiffnesses

Matrix stiffening is mediated by epithelial and/or stromal cells, which remodel their microenvironment, thereby promoting pathogenesis. Concomitantly, stiffening of the matrix impacts epithelial and mesenchymal cells. Since few 3D devices have been produced with controlled stiffness, the impact of ECM rigidity on intestinal and mesenchymal cells has been obtained using 2D substrates mimicking pathological colon stiffness. The matrix stiffness modulates the morphology and adhesion of myofibroblasts [63], promoting fibrogenesis through RhoA pathways [114] and stimulating the expression of prometastatic protein activin A [115]. Matrix stiffening also mediates the upregulation of ISC markers (CD133, ALDH-1 and LGR5) in HCT116 human colorectal cancer cells [116] and increases the production of MMP-7 in the T84 colon cancer cell line [117], both through the activation of the Yes-associated protein (YAP) and integrin β1/focal adhesion kinase (FAK) pathways. The role of the FAK signaling pathway in the substrate stiffness promotion of colon cancer progression has also been demonstrated in vitro and in vivo and seems to be induced by LOX-mediated collagen crosslinking [118]. Indeed, subcutaneous grafted cancer cells lines SW480 or HT29 expressing catalytically inactive LOX protein in nude mice demonstrated that LOX activity is necessary for cancer progression and an increase of tumor stiffness, and this effect is mediated through increased phosphorylation of FAK and the SRC tyrosine kinase.

The impact of matrix stiffening on CRC progression and on cancer stem cells (CSC) is now well-addressed [119]. However, little is known about its effect on the physiological ISC phenotype and its potential role on the tumorigenesis of ISC during chronic inflammation. Matrix stiffening has been demonstrated to induce a malignant phenotype, characterized by invasive behavior, the increased expression of protumoral genes such as estrogen receptor 1 α and the increased activation of the proliferative and migratory PI3K-AKT pathway in mammary epithelial cells MCF10a cultured in 3D in an alginate network containing laminin proteins [120]. Moreover, extensive evidence supports an important implication of epithelial-mesenchymal transition (EMT), a process leading to the loss of cell–cell adhesion and apical–basal polarity of epithelial cells, in inflammatory pathologies, as recently reviewed for CD [121], as well as in cancer progression, including colorectal cancer [122]. Thus, stiffening of the stroma should most likely promote an ISC switch from a physiological to a protumoral state, i.e., a transformation in CSC, yet further analyses should be done to decipher the molecular mechanisms involved.

#### 3.2.3. Deformability

##### Physiological Deformation Capacity of the Colon Epithelium

Deformability is the capacity of the colon epithelium to elongate and to shrink without tearing. The colon mucosa is subjected to two types of strains: peristalsis, i.e., repeated internal contractions necessary for stool progression, and shear stress, corresponding to the displacement of the food bowels at the surface of the epithelium, both exposing colorectal ECM and epithelium to major mechanical constraints [123]. From a histological perspective, scanning electron microscopy (SEM) studies on rat small intestine samples have shown that ECM fibers are organized as two interwoven arrays running diagonally around the crypt wall: one set clockwise and the other one set counter clockwise and oriented at a range of angle ± 30–50° [124]. It has been proposed that, during peristalsis, this diagonal orientation is essential to ensure the mucosa flexibility by modifying angles according to the degree of stretching [125]. In tumor tissues, the collagen network is remodeled with an increase in fiber alignment, resulting in increased tissue stiffness [104] and, probably, a modified deformability capacity. Indeed, if the evolution of resilience to peristalsis in pathological contexts is poorly described, peristalsis in patients suffering from IBD is decreased [126].

##### How to Induce Strain on Cell Cultures

Another step of the complexity of the production of a colon topography-mimicking structure concerns the deformation forces applied at the surface and the interstitial flow pressure in the inner matrix. Indeed, peristalsis allows food progression through contraction of the muscularis mucosae from the depths of the tissue while passage of the alimentary bolus induces shear stress and flow exchange at the surface of the epithelium.

Flow exchange is the easiest parameter to set up. Pioneer works used cells cultured on microporous membranes on a static system, i.e., the Transwell assay. It has been complexified by the introduction of a crypt-villus porous scaffold on the upper face of the membrane, thus allowing a topographic parameter introduction in the system [89,90,93]. To evolve from a static to a mobile flow, researchers took advantage of technological progress to introduce a microfluidic [95,127,128,129]. The addition of independent channels allows a medium perfusion at a controlled flow rate and measurement of ions, molecules and factors released by the cells at both the apical and basal sides. It also improves the survival and differentiation of the cells cultured on the device and allows the culture maintenance up to a month [95] and can be completed with microelectrodes for transepithelial electrical resistance measurements [130]. A new challenge has been raised by the combination of multi-chambers in a microfluidic device, allowing the creation of “human-on-chip” systems [131,132]. The different chambers of the device can be filled by cells from specific organs in order to optimize drug screening, therapeutic researches and adverse effects studies.

Mimicking the contraction of the intestinal muscle constitutes another difficulty. As early as the end of the 1990s, Basson et al. used a vacuum to induce the cyclic deformation of a collagen-coated membrane covered with Caco-2 cells, thus exposing the cultured epithelium-to-peristalsis motion [133,134]. Ingber’s team was the first to produce a more elaborate device, creating lateral mechanical constraints coupled to the microfluidic [135]. The device consists of three aligned compartments. Cells are cultivated in the middle one while the vacuum is applied to the side chambers. Ingber’s group also successfully used this device to culture human primary small intestinal cells [136].

The potentials complexifications of such systems were recently reviewed by Park et al. [137].

##### Importance of Deformation on Cell Phenotypes

In vivo, mechanical deformation of the intestine has an impact on the organ size and topography. Repeated stretches of the small intestine in a pig model resulted in an elongation of the intestinal segment but, also, in increased crypt depth and cell proliferation [138]. In vitro, a peristalsis-like motion and fluid flow promoted the spontaneous formation of crypt-villus structures on Caco-2 cells cultured on a flexible porous membrane coated with collagen type I and Matrigel, with a high integrity barrier [139]. It was further confirmed by Kasendra et al. using human primary intestinal epithelial cells plated on the upper chamber of Ingber’s device and subjected to cyclic deformation and constant flow for several days [136]. At a molecular level, the proliferation and differentiation of Caco-2 cells induced by repetitive strain involved proteins with tyrosine kinase activity, one of them being protein kinase C (PKC) [133,134]. Indeed, the PKC inhibitor calphostin C attenuates deformation-induced ^3^H-thymidin incorporation and alkaline phosphatase activity in Caco-2 cells [134]. One can suppose that such mechanisms should be similar in the ISC regulation of growth and differentiation; besides, investigations on peristalsis and fluid flow effects on stem cells are just getting started, presenting an opportunity for future research.

## 4. Conclusions

Characterizing ISC interactions with their environment is critical to understanding processes such as intestinal tissue establishment, renewal and, also, altered regeneration processes occurring during IBD or cancer initiation. Today, technological progress takes the lead for intestinal and colorectal physiological understanding. A major breakthrough during the last decade was the generation of intestinal organoids [140]. More specifically, human organoid models give great hope, as they represent an excellent tool to fill the gap between animal models and humans. Indeed, animal model are created by treating the animals with harmful substances or are generated by recreating the genetic alterations found in disease. However, one has to admit that they do not always recapitulate correctly the human disease. Regarding colorectal cancer, for instance, most transgenic mice models displaying the main mutations involved in the human adenocarcinoma sequence mainly develop tumors in the small intestine and only rarely in the colon. A main advantage of the organoids is that they are directly generated from the patient tissues, without knowing the specific genes involved. This is particularly interesting to study multigenic disorders such as IBD or cancer [141,142,143,144,145,146]. Three-dimensional colon organoid cultures recapitulate in vitro the heterogeneity of the intestinal tissue but do not fully reproduce the colon architecture. In cultures, the reconstitution of an ISC niche providing all the biochemical factors necessary for the promoting of either the ISC renewal or cell lineage differentiation is of high importance, depending on the studied process or the addressed question. The ECM is another crucial player, and today, more and more studies not only consider its biochemical composition but, also, its mechanical properties.

However, intestinal organoids cultivated in hydrogels in 3D cannot support the formation of signaling gradients and mechanical forces and, therefore, do not fully recreate the native microenvironment. To address these limitations, several studies reported the development of newly engineered culture systems to study the intestinal epithelium. In particular, recent advances in microfabrication technologies and biomaterials made possible the development of new 3D in vitro systems more physiologically relevant to study the intestinal epithelium. Most of these systems use the human colorectal adenocarcinoma cell line, Caco-2 cells that spontaneously differentiate into a polarized mature intestinal epithelium. In order to recreate the architecture of the intestinal epithelium, various 3D micropatterned scaffolds mimicking intestinal villi or crypts have been developed, mostly using molding or lithography technologies. These studies have clearly demonstrated the interplay between cell differentiation, polarization and the 3D microenvironment. Recently, a 3D model reproducing the crypt-villus architecture and biochemical gradients using micro-molded crosslinked collagen was developed to culture primary human intestinal cells. This model showed that 3D topology, in addition to other physical and chemical cues, is a key factor to promote cell differentiation and polarization. However, due to limitations in the resolution and/or complexity of the fabrication techniques, many of these 3D models still fail to fully recapitulate or accurately control the topology of crypt-villus compartments. However, to date, none of them takes into consideration the mechanical properties of the ECM. Stiffness appears trickier to achieve in part because of discrepancies regarding the measurement of the actual elastic modulus of the tissue. Finally, installing a constant stream at the apical pole of colon epithelial cells, at an accurate flow rate, and pairing it with physical stretching at the basal pole of the cells is still challenging. The last challenge, but not the least, will be to put each parameter together and accommodate primary ISC cultures.

Hopefully, within the future decade, the development of a microphysiological system providing spatial and temporal control of the cell microenvironment (automated microfluidic control, tuning of the matrix properties, topology, etc.) coupled to primary intestinal organoid and stromal cell cultures will certainly represent an amazing tool to investigate stromal cells, intestinal cells and cell–ECM interplays but, also, the intestinal barrier function, microbiota and alimentation/nutritional impact on these cells. The development of such a tool can help to understand whether or not normal crypt stem cells can alter their capacities to repair/regenerate under environmental pressure but, also, to envisage new diagnostic, follow-up and therapeutic approaches for patients presenting intestinal pathologies such as IBD or cancer, depending on the origin of the tissue (IBD or cancer resection) used to establish the primary cultures.

## Figures and Tables

**Table 1:** Summary of the basement membrane (BM) components and cellular integrin differential expressions along human small intestinal/colon tissues. COLL: collagen.

	Proteins	Bottom Crypt	Upper Crypt	Villus
BM	COLL4A1, COLL4A2	+	+	+
	COLL4A5	+	+	+
	COLL4A3, COLL4A4	−	−	−
	Perlecan	+	+	+
	Laminin α1β1γ1	−	−	+
	Laminin α2β1γ1	++	+	−
	Fibronectin	++	+	−
	Tenascin-C	−	+	++
Integrin	β1	+	+	+
	β4	+	+	+
	α1	−	+	−
	α2	+	+	−
	α3	−	+	+
	α4	−	−	−
	α5	−	−	−
	α6	+	+	+
	α7	−	+	−

**Table 2:** Summary of the fabrication processes for intestinal 3D scaffolds. CVD: chemical vapor deposition and pHEMA: poly(2-hydroxyethylmethacrylate). PED-GA: poly(ethylene) glycol diacrylate, PDMS: polydimethylsiloxane, PMMA: polymethyl methacrylate, PLGA: poly-lactic-glycolic acid and HUVEC: human umbilical vein endothelial cells.

Materials	Technology for Mold Creation	Scaffold	Dimensions	Cell Culture	Ref.
CVD pHEMA	CVD reactor	crypts-villi	pig small intestinal tissue	Caco-2	[92]
40% PEG-DA 700 + 30% acrylic acid + 250-μg/mL fibronectin + 0.1% Irgacure 819	stereolithography	crypts-villi	Villi: 500 μm in height, 150 μm in diameter at the top and 300 μm at the bottom. Crypt: 200 μm in deep, 50 μm in diameter	SW80, Caco-2	[87]
epoxy/PDMS/collagen	spin-coating and photolithography	crypts-villi	Villi: 477 µm in height, 170 µm in diameter Crypt: 132 µm in depth, 60 µm in diameter Total height of crypt/villus 609 µm	Human primary colonic cells	[93]
PMMA/PDMS/alginate/collagen	CO_2_ laser system	villi	565 µm in height	Caco-2	[90]
PMMA/PDMS/alginate/collagen or PEG-DA	laser ablation	villi	500 µm in height	Caco-2	[88]
PMMA/PDMS/alginate/PLGA-porogen	laser ablation	villi	500 µm in height	Caco-2 + bacteria	[91]
PMMA/PDMS/alginate/PLGA-porogen	laser ablation	Villi	500 µm in height	Caco-2 + mice primary colonic cells	[89]
two collagen-based bioink-laden	bioprinting	villi	183 ± 12 μm in diameter and 770 ± 42 μm in height	Caco-2 + HUVECs	[94]
epoxy/PDMS/collagen	spin-coating and photolithography	crypts	430 µm in deep, 125 µm in diameter at the top, 200 µm spacing	human primary colonic cells	[86]
Sukhoi SU-8/PDMS + fibronectin	photolithography	crypts	50, 100, and 500 µm in diameter, 50 µm spacing, 120 µm in depth	Caco-2	[85]
Matrigel/Collagen type I	Laser ablation	crypts	75 µm in diameter at the top, 50 µm in diameter at the bottom, 170 µm in depth	Mouse and human primary intestinal stem cells	[95]

**Table 3:** Summary of sample preparations and instruments used to determine the Young modulus (YM) in normal and inflamed or cancerous colons and intestines. AFM: atomic force microscopy.

Organ	*n*	Sample Preparation	YM (kPa)	Instrument	Ref.
Normal colon	3	Cryo-sectioned samples section, measurement on collagen-rich strictures.	mean 0.8 ± 0.4	AFM	[104]
Colon carcinoma	3		mean 2.4 ± 1.83 (0.9–4.4)		
Normal colon	106	Fresh sections longitudinally opened with mucosal side upward.	median 0.936 (0.374–7.33)	Tactile sensor	[105]
Colon carcinoma	106		median 7.51 (1.08–68)		
Unaffected colon	13 from 1 patient	Stored in ice, tested within 4 h of isolation. Opened with mucosa upward.	mean 0.698 ± 0.463	Custom-built multiscale indenter	[64]
Inflamed colon	31 from 3 patients		mean 1.143 ± 0.488		
Unaffected ileum	21 from 5 patients		mean 0.641 ± 0.342		
Inflamed ileum	12 from 4 patients		mean 0.991 ± 0.379		
Unaffected small intestine	11	Fresh 1-cm^2^ section.	median 2.6	Microelastometer	[63]
Crohn’s Disease	11		median 16.7		
